# Platelet-rich plasma as a promising bioscaffold for enhancing peripheral nerve regeneration: An experimental study in a rat sciatic nerve model

**DOI:** 10.14440/jbm.2025.0083

**Published:** 2025-02-19

**Authors:** Sofija Pejkova, Velimir Stojkovski, Gordana Georgieva, Boris Aleksovski, Sofija Tusheva, Blagoja Srbov, Elena Rafailovska, Stefania Azmanova Mladenovska, Katerina Jovanovska, Bisera Nikolovska, Boro Ilievski, Boro Dzonov, Trpe Ristoski, Anita Petrushevska, Icko Gjorgoski

**Affiliations:** 1 University Clinic for Plastic and Reconstructive Surgery, Faculty of Medicine, University Ss Cyril and Methodius, Skopje 1000, North Macedonia; 2 Faculty of Veterinary Medicine, University Ss Cyril and Methodius, Skopje 1000, North Macedonia; 3 Institute for Biology, Faculty of Natural Sciences and Mathematics, University Ss Cyril and Methodius, Skopje 1000, North Macedonia; 4 Institute for Pathology, Faculty of Medicine, University Ss Cyril and Methodius, Skopje 1000, North Macedonia

**Keywords:** Platelet-rich plasma, Peripheral nerve regeneration, Nerve repair, Experimental study, Surgical techniques

## Abstract

**Background:**

Despite advancements in surgical treatments, impairments persist after peripheral nerve injuries, prompting a shift in research toward the microenvironment of injured axons. Platelet-rich plasma (PRP), rich in growth factors and derived from autologous blood, emerges as a potential candidate to accelerate nerve healing.

**Objective:**

This study investigated the role of PRP in enhancing peripheral nerve regeneration using a rat sciatic nerve model (*n* = 8) in female Wistar rats.

**Methods:**

A transected sciatic nerve model was created, with both hindlimbs repaired through end-to-end neurorrhaphy. PRP, prepared from the blood of a healthy Wistar rat, was applied to one hindlimb. Functional recovery was assessed using sciatic indices. At the 20-week time point, histological evaluations were performed to compare PRP-treated hindlimbs with control ones. Statistical analysis was conducted to compare the results between the two groups using three different calculations for specific parameters.

**Results:**

Walking track-based sciatic functional index (SFI) showed an improvement of 66.0%, 47.8%, and 71.6% (*p* < 0.05). Video analysis-based SFI revealed a 36.7% and 27.3% improvement (*p* < 0.05). Static sciatic index calculations indicated an improvement of 19.4% for vertical standing and 26.7% for standing on all four limbs (*p* < 0.001). Histopathological analysis showed a reduction in inflammation, a decrease in fibrosis, and the absence of macrophages in the sciatic nerves of the experimental group. Muscle specimens from the PRP-treated group exhibited fewer macrophages and significantly less fibrosis (*p* < 0.05). Overall, PRP treatment significantly improved all functional indices.

**Conclusion:**

This study demonstrated PRP’s utility in promoting peripheral nerve regeneration, highlighting its potential for both fundamental research and clinical applications.

## 1. Introduction

Peripheral nerve injuries (PNI) are a significant medical challenge, often resulting in permanent disability and reduced quality of life. Despite advances in conventional treatment, including the gold standard of direct, tension-free microsurgical repair, functional recovery remains difficult to achieve.^1^ Recent advancements in neurobiology have shifted the focus of nerve regeneration research from the mechanical aspects of surgical repair to the microenvironment of the damaged axon. Growth factors, such as nerve growth factor, insulin-like growth factor (IGF), platelet-derived growth factor (PDGF), and fibroblast growth factor have been investigated as potential therapeutic strategies to promote nerve regeneration.^2^-^5^ These factors, which possess mitogenic and chemotactic properties, are stored in platelet alpha-granules, indicating that platelets play a key role in tissue healing.^6^,^7^

One promising agent is platelet-rich plasma (PRP), an autologous blood product containing high concentrations of platelets in a small volume of plasma. PRP maintains physiological levels of coagulation factors, which are essential for hemostasis. Beyond its role in promoting healing in various tissues, such as skin, tendon, ligament, muscle, and bone, PRP also exhibits adhesive properties.^8^ The PDGFs found in PRP enhance healing by accelerating regeneration and improving tissue quality.^8^ Several studies have shown that PRP can significantly enhance nerve regeneration following PNI.^9^ In addition, activated PRP facilitates structural guidance for injured nerves by forming a fibrin scaffold around them, supporting the healing process. Based on these findings, we hypothesized that local application of PRP, in conjunction with conventional microsurgical repair, may enhance recovery in a rat sciatic nerve model by promoting more effective functional recovery and accelerated nerve growth, facilitated by the numerous growth factors in PRP.^10^,^11^

## 2. Materials and methods

### 2.1. Animals

Eight healthy female Wistar rats (weight: 200 – 250 g; age: 14 – 16 weeks) were obtained from the Vivarium of the Faculty of Natural Sciences and Mathematics, University Ss Cyril and Methodius, Skopje, North Macedonia, and used in this study. The animals were housed under standard environmental conditions (temperature: 20 ± 2°C; relative humidity: 55 ± 10%; 12-h light/dark cycle). They were fed a standard pellet diet (20% protein, 30% carbohydrate, 9% lipid, 2.5% cellulose, 10% water, and 310 kcal energy) and provided with water *ad libitum*. All experiments were performed in accordance with current ethical standards, following the International Guiding Principles for Biomedical Research Involving Animals, as issued by the Council for the International Organizations of Medical Sciences. The study also complied with the European Community guidelines for laboratory animal use and care (EEC Directive of 1986; 86/609/EEC). Anesthesia was induced using xylazine (10 mg/kg; intraperitoneal; Interchemie, the Netherlands) and ketamine (100 mg/kg; intraperitoneal; Alfasan, the Netherlands), according to the EEC Directive 86/609/EEC. After the procedure, the animals were sacrificed using a standard laparotomy technique.

### 2.2. Preparation of PRP

Blood was collected from the dorsal vein of a healthy adult Wistar rat using a butterfly needle (18G) and a special Arthrex Autologous Conditioned Plasma double syringe, with the addition of 1 ml of citrate-phosphate-dextrose solution with adenine anticoagulant.^12^,^13^ The concentration of platelets in the whole blood was measured using an automatic hematology analyzer, which estimated the platelet count at 232 × 10^9^ platelets/μL. The syringe was sealed with a red cap and placed in a Rotofix 32 A Hettich Zentrifugen centrifuge (Andreas Hettich GmbH & Co. KG, Germany), where it was centrifuged at 150 g for 5 min. The PRP was then separated from the rest of the blood, and it was extracted from the syringe by slowly and carefully pulling the plunger upward, following the manufacturer’s instructions. The PRP was transferred to a sterile 1 cm^3^ plastic syringe. The platelet concentration of the PRP, as measured by the automatic hematology analyzer, was estimated to be 458 × 10^3^ platelets/μL. To activate the platelets, a freeze-thaw method was employed.

### 2.3. Surgical procedures

A transected sciatic nerve model was created in all eight female Wistar rats. All surgical interventions were performed by the same surgeon under aseptic working conditions, using general anesthesia and microsurgical instruments under magnification. The rats were positioned and immobilized on the operating table, and a surgical incision was made in the posterior gluteal region, extending from the spine to the popliteal area. For better exposure, a long incision was made down to the subcutaneous tissue. The sciatic nerve was then exposed through blunt dissection of the biceps femoris muscle, from its exit point on the spinal cord to the first distal branch. After exposure, the sciatic nerves of both hindlimbs were transected, and immediate repair was performed using end-to-end (E-E) neurorrhaphy with 8/0 nylon sutures under ×10 magnification. A volume of 0.15 – 0.2 mL of pre-prepared PRP was locally applied to the right hindlimb after the E-E neurorrhaphy. The left hindlimb, which received E-E neurorrhaphy only, served as the control. After the procedure, the exposed soft tissues were closed in anatomical layers.

### 2.4. Functional indices

The recovery of the limb after sciatic nerve injury was observed over a 20-week (5-month) post-operative period. Functional recovery of the sciatic nerve was comprehensively assessed by using three different methodological approaches, resulting in a total of seven indices. These methodologies included:

(i) Walking tracks measurements: footprints were analyzed during movement using three different calculations of the sciatic functional index (SFI), as described by de Medinaceli *et al.*,^14^ Carlton *et al.*,^15^ as well as Bain *et al*.^16^
(ii) Video analyses of walking: two different SFI calculations were used, based on the methods of Carlton and Goldberg^15^ and Bain *et al*.^16^
(iii) Photo analyses of standing: the static sciatic index (SSI) was estimated according to Bervar,^11^ in two different postures: standing on hindlimbs only (vertical standing) and standing on all four limbs.

The variables required to calculate the SFI and SSI included measurements of foot length (i.e., print length, defined as the distance from the heel to the third toe), total spreading (i.e., the maximum distance between the first and the fifth toes), intermediary toe spreading (the distance between the second and fourth toes, and the distance to the opposite foot).

### 2.5. Tissue preparation and sampling for histological analysis

At the 20-week time point, the sciatic nerves and gastrocnemius muscles from all eight rats were harvested for histological evaluation to determine any differences between the untreated control hindlimbs and the PRP-treated hindlimbs. The tissue samples were embedded in paraffin, sectioned on a Leica HistoCore BIOCUT microtome (United States), processed with a Leica TP 1020 tissue processor (United States), and stained with hematoxylin and eosin. The nerve samples were cut in both longitudinal and partial transverse sections, while the muscles were cut transversely.

With the sciatic nerve samples, the following parameters were assessed: the presence of inflammatory cells in the nerve bundles, vacuolization of the endoneurium, and the degree of nerve fibrosis (classified as mild, moderate, or severe). These parameters were evaluated by analyzing 15 fields at high power magnification (×400).

In the gastrocnemius muscle samples, the presence of macrophages and other inflammatory cells in the muscle bundles was determined by analyzing 15 fields at ×400 magnifications. In addition, the degree of fibrosis was classified as mild, moderate, or severe.

### 2.6. Statistical analyses

Statistical analyses were performed using IBM SPSS Statistics^®^ 21 (United States) and GraphPad Prism (version 9, United States). All categorical variables are expressed as percentages, and the Chi-square test was used to assess the significance of differences in frequencies among the groups. Quantitative variables are presented as mean ± standard deviation, and comparisons of central tendencies between the two groups were made using an unpaired two-tailed *t*-test. The statistical analysis of the functional indices over time also included calculations of the area under the curve (AUC) for the trendlines depicting temporal changes. A *p* < 0.05 (marked as *) was considered statistically significant, and a *р* < 0.001 was considered highly significant (marked as ***).

## 3. Results

### 3.1. Functional indices

The comprehensive evaluation of the sciatic nerve functional recovery, based on three different methodological approaches, resulted in a total of seven indices. Key comparisons of functional recovery between the left hindlimbs (control) and the right PRP-treated hindlimbs were made at one week, 10 weeks, and 20 weeks post-operatively. To assess overall improvement, we considered the full temporal trend of functional recovery in the PRP-treated hindlimbs compared to the untreated control group over the 20-week post-operative period. Therefore, AUC comparisons were considered the most valid measure for evaluating the overall temporal changes in recovery.

#### 3.1.1. Walking track-based SFI

**Figure 1:**
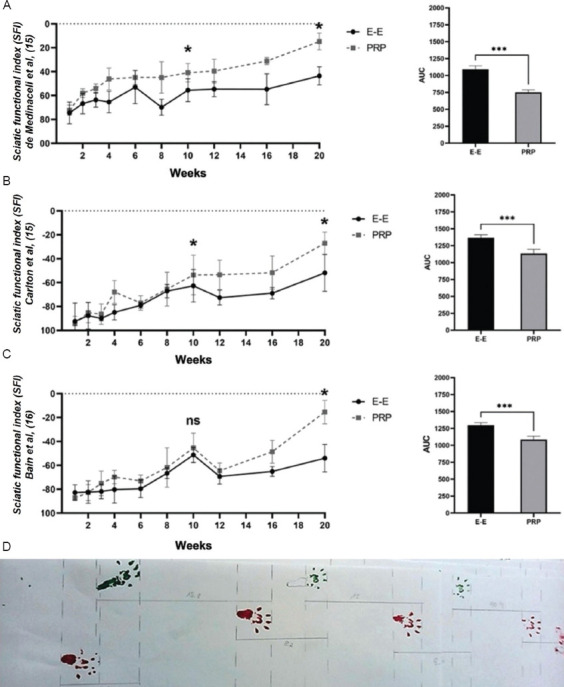
Sciatic functional index (SFI) during movement, based on measurements taken from walking tracks. (A) SFI, calculated according to de Medinaceli *et al*.’s method.^14^ (B) SFI, calculated according to Carlton and Goldberg’s method.^15^ (C) SFI, calculated according to Bain *et al*.’s method.^16^ (D) Calculation and measurements from the obtained footprints. Notes: *p<0.05; ***p<0.001. *p*-values were estimated using an unpaired two-tailed *t*-test. Abbreviations: AUC: Area under the curve; E-E: End-to-end repair (control group); ns: Non-significant; PRP: Platelet-rich plasma-treated (experimental group).

The SFI during movement, based on measurements of walking track footprints (Figure 1D), was calculated using three different methods used by: de Medinaceli *et al*.^14^ (Figure 1A), Carlton and Goldberg^15^ (Figure 1B), and Bain *et al*.^16^ (Figure 1C and Figure 1). One week post-operatively, none of the three SFI calculations showed significant differences in SFI calculations between the PRP-treated and untreated hindlimbs. However, at 10 weeks post-operatively, significant improvements were detected in two of the SFI calculations: a 26.4% improvement (Figure 1A) and a 14.5% improvement (Figure 1B) in the PRP-treated hindlimbs (*p* < 0.05), whereas the SFI calculation according to Bain *et al*. (Figure 1C) did not show significant differences. At 20 weeks post-operatively, significant improvements were observed in all three SFI calculations, with 66.0% (Figure 1A), 47.8% (Figure 1B), and 71.6% (Figure 1C) improvements in the PRP-treated hindlimbs (*p* < 0.05). AUC comparisons of the temporal trends between the PRP-treated and untreated hindlimbs showed significant improvements in all three SFI calculations. Specifically, Medinaceli *et al*.’s calculation showed a 31.2 % improvement (Figure 1A; t = 12.0; *p* = 0.0001), Carlton and Goldberg’s calculation exhibited a 17.0% improvement (Figure 1B; t = 6.81; *p* = 0.001), and Bain *et al*.’s calculation yielded a 16.3% improvement (Figure 1C; t = 7.80; *p* = 0.0001).

#### 3.1.2. Video analysis-based SFI

**Figure 2:**
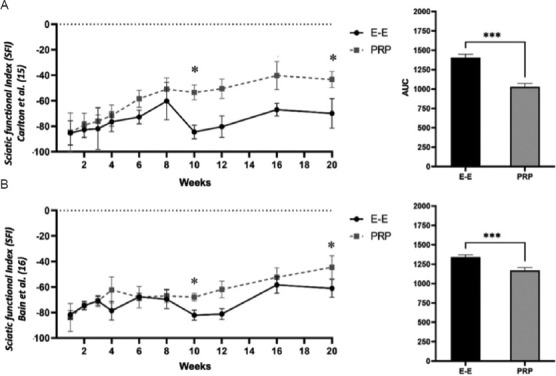
Sciatic functional index (SFI) during movement, based on video analyses of walking. (A) SFI, calculated according to Carlton and Goldberg’s method.^15^ (B) SFI, calculated according to Bain *et al*.’s method.^16^ Notes: *p < 0.05; ***p < 0.001. *p*-values were estimated using an unpaired two-tailed *t*-test. Abbreviations: AUC: Area under the curve; E-E: End-to-end repair (control group); PRP: Platelet-rich plasma-treated (experimental group).

Figure 2 shows the SFI based on video analysis of walking, calculated using the methods of Carlton and Goldberg (Figure 2A) and Bain *et al*. (Figure 2B). One week post-operatively, none of the SFI calculations showed significant differences between the PRP-treated and untreated hindlimbs. Nevertheless, by week 10, SFI calculated according to Carlton and Goldberg showed a significant improvement in the PRP-treated limbs (E-E: PRP, 36.7%; *p* < 0.05), and at week 20, a further improvement was observed (E-E: PRP, 40.0%; *p* < 0.05). In contrast, SFI calculated according to Bain *et al*. showed a significant improvement only at week 20 post-operatively (E-E: PRP, 27.3%; *p* < 0.05). AUC comparisons of the video-based SFI calculations revealed significant differences between the temporal trends of recovery in the PRP-treated and untreated hindlimbs. Carlton and Goldberg’s calculation showed a 28.6% improvement (Figure 2A; t = 13.57; *p* = 0.0001), while Bain *et al*.’s calculation showed a 12.8% improvement (Figure 2B; t = 8.10; *p* = 0.0001).

#### 3.1.3. SSI based on photo analysis

**Figure 3:**
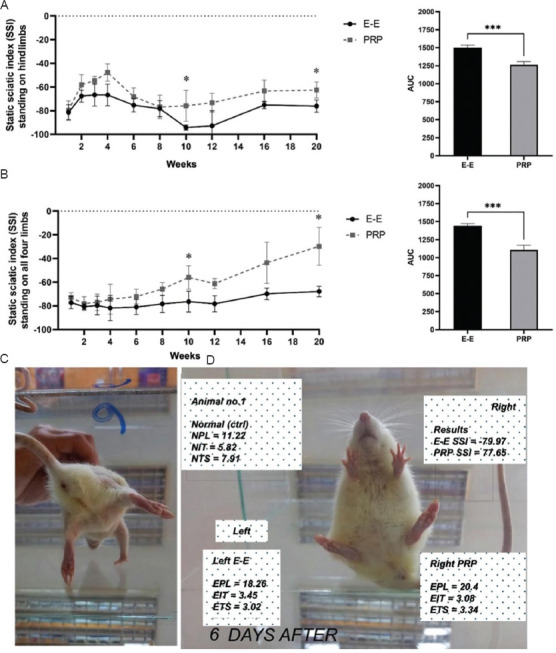
Static sciatic index (SSI) based on photo analyses of standing. (A) SSI, based on photo analysis of standing on hindlimbs only (vertical standing). (B) SSI, based on photo analysis of standing on all four limbs. (C) Position for analysis of “vertical standing” on hindlimbs only. (D) Calculation of the SSI based on standing on all four limbs. Notes: *p < 0.05; ***p < 0.001. *p*-values were estimated using an unpaired two-tailed *t*-test. Abbreviations: AUC: Area under the curve; ctrl: Control; EIT: Experimental intermediary toe spreading; EPL: Experimental print length; ETS: Experimental toe spreading; E-E: End-to-end repair (control group); NIT: Normal intermediary toe spreading; NPL: Normal print length; NTS: Normal toe spreading PRP: Platelet-rich plasma-treated (experimental group).

Figure 3 presents the SSI based on photo analysis of standing on hindlimbs only (i.e., vertical standing; Figure 3A) and standing on all four limbs (Figure 3B). Over time, the vertical standing SSI showed significant improvement at week 10 (E-E: PRP, 19.4%; *p* < 0.001) and at week 20 (E-E: PRP, 19.5%; *p* < 0.001). The SSI for standing on all four limbs showed even greater improvements in the PRP-treated hindlimbs, with a 26.7% improvement at week 10 (*p* < 0.001) and a 56.1% improvement at week 20 (*p* < 0.001). Moreover, both SSI indices revealed significant differences in the AUC of the time-course curves. When standing on all four limbs, the PRP-treated group showed a 23.2% overall improvement (t = 10.38; *p* = 0.0001), while standing on hindlimbs only exhibited a 15% improvement (t = 9.24; *p* = 0.0001).

### 3.2. Histopathological findings

**Figure 4:**
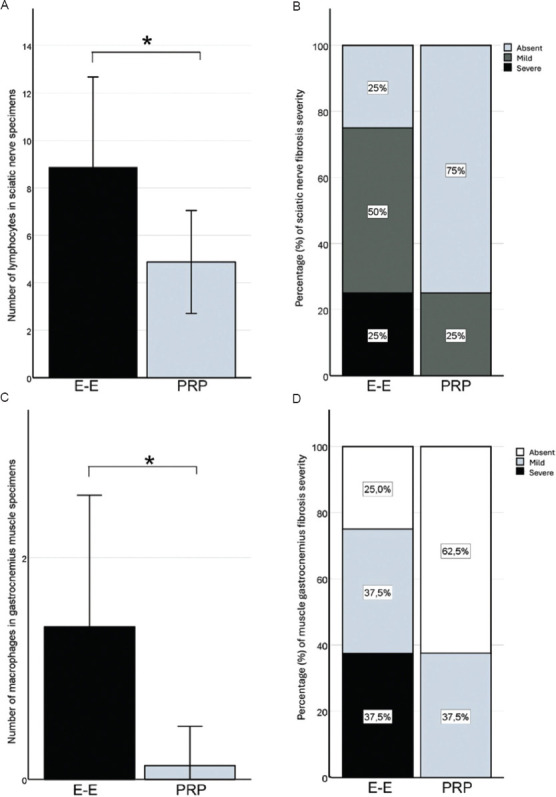
Histopathological evaluation. (A) Number of lymphocytes in the sciatic nerves. (B) Severity of sciatic nerve fibrosis. (C) Number of macrophages in the bundles of the gastrocnemius muscle. (D) Severity of gastrocnemius muscle fibrosis. Notes: *p < 0.05. *p*-values were estimated using an unpaired two-tailed *t*-test. Abbreviations: E-E: End-to-end repair (control group); PRP: Platelet-rich plasma-treated (experimental group).

**Figure 5:**
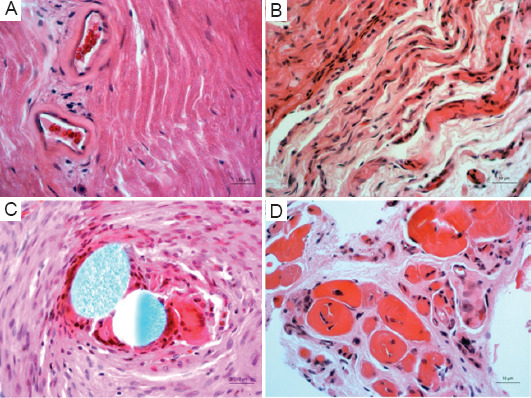
Microphotographic evidence of pathological findings in the untreated end-to-end hindlimbs under 400× magnifications. (A) Massive presence of numerous lymphocytes in the sciatic nerve. Scale bar: 10 μm. (B) Severe fibrosis observed in the sciatic nerve of the untreated hindlimbs. Scale bar: 10 μm. (C) Granuloma detected in the sciatic nerve of the untreated hindlimbs. Scale bar: 10 μm. (D) Severe muscle fibrosis in the untreated hindlimbs. Scale bar: 10 μm.

Figures 4 and 5 illustrate the key histopathological findings from this study. The sciatic nerves of PRP-treated hindlimbs exhibited a significantly lower number of lymphocytes (t = 6.70; *p* = 0.021; Figure 4A) and a reduced frequency of moderate fibrosis (only 25%; χ^2^ = 4.35; *p* = 0.037; Figure 4B). Contrarily, the untreated control hindlimbs showed notable pathological findings, including moderate fibrosis in 50% and severe fibrosis in 25% of the analyzed nerve samples (Figures 4B and 5B). In addition, the untreated group displayed macrophages within the nerve bundles. Importantly, neither severe fibrosis nor the presence of macrophages was observed in any of the sciatic nerve samples from the PRP-treated group. Moreover, granulomas composed of aggregated macrophages were detected in the untreated E-E control group (Figure 5C), suggesting chronic inflammation. The significantly lower number of lymphocytes and the absence of macrophages in the sciatic nerve specimens of the PRP-treated hindlimbs suggest a reduced immune response inflammation, which could foster a more favorable environment for nerve regeneration. In contrast, the enhanced fibrosis and the presence of granulomas in the untreated E-E hindlimbs suggest poorer healing conditions, as excessive immune responses and chronic inflammation can hinder nerve recovery. Regarding vacuolization of the sciatic nerve endoneurium, statistical analyses revealed no significant differences between the two groups, suggesting that PRP treatment did not exacerbate cellular stress or nerve damage.

Histological examination of the gastrocnemius muscle preparations showed a significantly lower number of macrophages in the PRP-treated hindlimbs (Figure 4C; t = 2.85; *p* = 0.013), as well as a complete absence of muscle fibrosis in 62.5% of the samples (Figure 5D). In contrast, only 25% of the untreated hindlimb samples displayed no fibrosis (χ^2^ = 2.29; *p* = 0.038). Severe fibrosis was evident only in the untreated hindlimbs (Figure 5D), with 37.5% of the samples showing distinct fibrosis (Figure 4D). No other inflammatory cells were detected in the muscle tissue. Taken together, these histopathological findings suggest that PRP treatment promotes sciatic nerve regeneration by reducing immune response, decreasing muscle and nerve fibrosis, and controlling inflammation in the nerve, without contributing to increased cellular stress or damage.

## 4. Discussion

To assess the effect of PRP on nerve regeneration, we utilized multiple methods for evaluating functional recovery, including walking track analysis, video analysis of walking, and SSI measurement. All of these tests consistently demonstrated significant improvements in the PRP-treated hindlimbs compared to the untreated control group.

Initially, during the 1^st^ post-operative week, no significant differences were observed between the groups in terms of functional recovery. However, as the study progressed, several interesting results emerged. For example, at 10 weeks post-operatively, the PRP-treated group showed improvements in various indices, with a 26.4% improvement based on the SFI calculation using walking track data. Other indices, such as the SFI calculation by Carlton and Goldberg, showed a 36.7% improvement according to video-based analysis. These results suggest that PRP exerts a positive effect on early functional recovery.

Remarkably, all functional indices demonstrated significant improvements in the PRP-treated hindlimbs at the 20-week mark. These included a 66.0% improvement in walking track-based SFI on the basis of Medinaceli *et al*.’s calculation, 47.8% using Carlton and Goldberg’s, and 71.6 % based on Bain *et al*.’s, compared to the control group.

The AUC comparisons for temporal changes in recovery further supported the overall efficacy of PRP treatment. In all seven calculations of SFI and SSI, we observed considerable improvements over the entire post-operative period.

Histopathological findings also supported these functional improvements. The sciatic nerves of PRP-treated hindlimbs showed a lower number of lymphocytes, a reduced frequency of fibrosis, and the absence of macrophages compared to the untreated control group. In contrast, the untreated control hindlimbs exhibited moderate-to-severe fibrosis, along with infiltration of inflammatory cells, including macrophage aggregates, suggesting chronic inflammatory processes.

In addition, the lower lymphocyte count and the absence of macrophages in the sciatic nerves of PRP-treated samples indicate a reduced immune response and less inflammation, creating a more favorable environment for nerve regeneration. On the other hand, the higher degree of fibrosis, particularly the severe type in the control group, may imply suboptimal healing conditions, possibly due to excessive immune responses or persistent inflammation.

These results suggest that PRP application may enhance peripheral nerve repair. Potential mechanisms include the control of inflammation, enhancement of growth factors, and the creation of a favorable environment for nervous system healing. Moreover, activated PRP creates a fibrin scaffold that mimics natural extracellular matrices, thereby facilitating cell adhesion, migration, and proliferation, while also providing structural support. Beyond these functions, the slow release of growth factors from the PRP within the scaffold may contribute to long-term recovery.

### 4.1. Functional indices

To evaluate the impact of PRP on nerve regeneration, we employed several methodological approaches to assess functional recovery, including walking track analysis, video analysis of walking, and SSI measurement. These analyses consistently demonstrated significant improvements in the PRP-treated hindlimbs compared to the untreated control group.^17^

Walking is a coordinated activity involving sensory input, motor response, and cortical integration;^18^ therefore, walking track analysis (SFI) is a comprehensive test for functional recovery. The present study showed that PRP treatment improved functional recovery of the sciatic nerve throughout the study period, as evidenced by improvements in all SFI indices measured. In addition, PRP treatment led to improvements in SSI, both in vertical standing and standing on all four limbs. Interestingly, the time-course curves for all seven indices followed similar trends.

Initially, the 1^st^ post-operative week did not reveal significant differences in functional recovery between the two groups. However, as the study progressed, notable differences emerged. At 10 weeks post-operatively, the PRP-treated group exhibited improvements in various indices, including a 26.4% (Medinaceli *et al*.’s calculation) and 14.5% (Carlton and Goldberg’s calculation) improvement in walking track-based SFI, along with a 36.7% improvement in video-based SFI calculated according to the method of Carlton and Goldberg. These findings suggest that PRP application positively impacted early functional recovery.

Remarkably, at the 20-week mark, all functional indices demonstrated significant improvements in the PRP-treated hindlimbs. These included a 66.0%, 47.8%, and 71.6% improvement in walking track-based SFI, based on Medinaceli *et al*.’s, Carlton and Goldberg’s, and Bain *et al*.’s method, respectively, compared to the control group.

Comparisons of the AUC for the temporal changes in recovery further supported the overall effectiveness of PRP treatment. The AUC comparisons indicated that all seven SFI and SSI calculations showed marked and significant improvements throughout the entire post-operative period in the PRP-treated group.

### 4.2. Histopathological findings

Histopathological analysis corroborated the functional recovery data. The PRP-treated group exhibited a lower number of lymphocytes, reduced fibrosis, and the absence of macrophages in the sciatic nerves compared to the untreated group. In contrast, the untreated control hindlimbs displayed signs of moderate-to-severe fibrosis, inflammation, and the presence of macrophages.

The reduced number of lymphocytes and the absence of macrophages in the PRP-treated group suggest a diminished immune response and decreased inflammation in the nerve. These changes may foster a more favorable environment for nerve regeneration. In contrast, the presence of fibrosis – particularly severe fibrosis – in the control group’s nerve samples indicates less optimal healing conditions, likely due to excessive immune responses and chronic inflammation.

Histopathological analysis of the gastrocnemius muscles also revealed favorable outcomes in the PRP-treated group. These muscles exhibited significantly fewer macrophages and less fibrosis, with some samples showing no fibrosis at all. In contrast, the untreated hindlimbs displayed severe fibrosis and a higher presence of macrophages within the muscle bundles.

### 4.3. Interpretation of findings

These results collectively suggest that PRP application positively influences peripheral nerve regeneration. PRP’s ability to modulate inflammation, promote the release of growth factors, and create a favorable environment for nerve repair likely contributes to its therapeutic potential. The fibrin scaffold formed by PRP activation may also play a pivotal role by mimicking the natural extracellular matrix, thus supporting cell adhesion, migration, and proliferation. In addition, the gradual release of growth factors from PRP within this scaffold can sustain the prolonged process of nerve regeneration.

### 4.4. Previous research and clinical implications

It is well-established that PRP can aid in the repair of peripheral nerves.^19^,^20^ The fibrinogen-containing growth factors in PRP activate cell attachment, migration, and proliferation by synthesizing fibrous scaffolds imitating blood clots, which act as natural extracellular matrices.^21^,^22^ Moreover, activated platelets secrete various cytokines required by nerves during the reconnection process of healing.^23^-^25^ Some researchers have leveraged this finding in neurogenic therapeutic efforts. For example, Yuan *et al*. demonstrated that PRP could control inflammation while accelerating nerve repair through a gel created with PRP.^26^ Furthermore, surgical operations were only successful after several animal trials showed positive results regarding peripheral nerve healing. In these studies, nerves were damaged (e.g., cut with a knife or exposed to acid burns) and then sutured PRP-treated sutures, which were removed after three days.^27^,^28^ PRP also stimulates Schwann cell proliferation due to IGF-1 and PDGF with two B subunits (PDGF-BB), promoting axonal regeneration along injured nerve tracts and hence aiding in recovery.^29^ However, these treatments must await further research in humans, as not all human physiological responses can be accurately mimicked in animal models. Moreover, many aspects of human biology remain poorly understood, making it critical to carefully assess the potential outcomes before widespread application. In addition, scientists have found that the concentration levels of PRP influence Schwann cell behavior, implying that optimizing PRP concentrations could enhance therapeutic outcomes. Finally, methods such as ultrasound-guided PRP injection may offer faster healing for damaged nerves, providing a promising alternative approach.^30^-^32^ These diverse findings underscore the multifactorial nature of PRP treatment in PNI, which could lead to new therapeutic options both in clinical practice and basic scientific research, further emphasizing its significance.

### 4.5. Challenges and future directions

Despite these promising results, several challenges remain. One notable challenge is the need for standardization in PRP preparation and application. Variability in these processes can affect therapeutic outcomes, emphasizing the importance of consistent protocols to ensure reliable and effective treatments.

## 5. Conclusion

The promising findings of this study include significant improvements in various measures of functional recovery following PNI treated with PRP. A range of methodological strategies were employed, including walking track analysis, video analysis of walking, and SSI measurement.

The results from our study provide valuable insights into the potential use of PRP as an adjuvant therapy for conventional microsurgical repair, leading to improved functional outcomes following PNI. However, it is essential to establish standardized methods for preparing and applying PRP, as variations in these processes may impact their therapeutic efficacy.

Overall, interest in the potential of PRP in regenerative medicine has been growing, particularly with respect to its application in nerve regeneration. This increasing interest should stimulate further investigations into the effects of PRP on nerve regeneration, ultimately contributing to better patient outcomes in the treatment of PNI.

## Data Availability

The data will be made available to readers in a database upon request to the corresponding author.
